# 1-[(*Z*)-8-(4-Chlorophenoxy)-3-(2,4-di­fluorophenyl)-4-oxaocta-2-en-2-yl]-1*H*-1,2,4-triazol-4-ium nitrate

**DOI:** 10.1107/S1600536811038761

**Published:** 2011-09-30

**Authors:** Jian-long Chen, Kai Wang, Yan Shen, Li-li Ren, Guo-guang Chen

**Affiliations:** a College of Pharmaceutical Science, Nanjing University of Technology, Xinmofan Road No.5 Nanjing, Nanjing 210009, People’s Republic of China; b Jiangsu Engineering Technology Research Center of Polypeptide Pharmaceuticals, College of Life Science and Pharmaceutical Engineering, Nanjing University of Technology, Xinmofan Road No.5 Nanjing, Nanjing 210009, People’s Republic of China

## Abstract

In the title compound C_21_H_21_ClF_2_N_3_O_2_^+^·NO_3_^−^, the triazole ring makes dihedral angles of 40.7 (3) and 30.2 (4)° with the 4-chloro­pheny and 2,4-difluoro­phenyl rings, respectively. The cation adopts a *Z*-configuration about the C=C double bond which links the triazole ring to the 4-chloro­phen­oxy unit *via* a but­yloxy chain. In the crystal, the cations and the anions are linked by N—H⋯O, C—H⋯O and C—H⋯F hydrogen bonding.

## Related literature

For the anti­fungal activity of related compounds, see: Jeu *et al.* (2003 ▶). For details of the synthesis, see: Ludwig & Kurt (1985 ▶). For a related structure, see: Kurt *et al.* (1987 ▶).
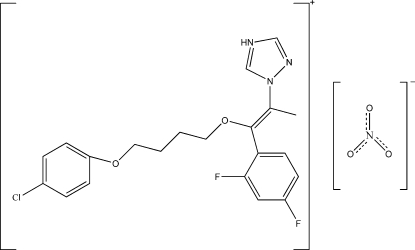


## Experimental

### 

#### Crystal data

C_21_H_21_ClF_2_N_3_O_2_^+^·NO_3_^−^
*M* *_r_* = 482.87
Monoclinic, 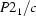

*a* = 7.9580 (16) Å
*b* = 31.635 (6) Å
*c* = 9.1850 (18) Å
β = 97.29 (3)°
*V* = 2293.6 (8) Å^3^
*Z* = 4
Mo *K*α radiation
μ = 0.22 mm^−1^
*T* = 293 K
0.30 × 0.10 × 0.10 mm

#### Data collection

Enraf–Nonius CAD-4 diffractometer
Absorption correction: ψ scan (North *et al.*, 1968 ▶) *T*_min_ = 0.936, *T*_max_ = 0.978
4521 measured reflections
4216 independent reflections
1952 reflections with *I* > 2σ(*I*)
*R*_int_ = 0.092
3 standard reflections every 200 reflections  intensity decay: 1%

#### Refinement

*R*[*F* ^2^ > 2σ(*F* ^2^)] = 0.066
*wR*(*F* ^2^) = 0.140
*S* = 1.00
4216 reflections
298 parameters
H-atom parameters constrained
Δρ_max_ = 0.20 e Å^−3^
Δρ_min_ = −0.24 e Å^−3^

### 

Data collection: *CAD-4 EXPRESS* (Enraf–Nonius, 1994 ▶); cell refinement: *CAD-4 EXPRESS*; data reduction: *XCAD4* (Harms & Wocadlo, 1995 ▶); program(s) used to solve structure: *SHELXS97* (Sheldrick, 2008 ▶); program(s) used to refine structure: *SHELXL97* (Sheldrick, 2008 ▶); molecular graphics: *SHELXTL* (Sheldrick, 2008 ▶); software used to prepare material for publication: *PLATON* (Spek, 2009 ▶).

## Supplementary Material

Crystal structure: contains datablock(s) global, I. DOI: 10.1107/S1600536811038761/pv2445sup1.cif

Structure factors: contains datablock(s) I. DOI: 10.1107/S1600536811038761/pv2445Isup2.hkl

Supplementary material file. DOI: 10.1107/S1600536811038761/pv2445Isup3.cml

Additional supplementary materials:  crystallographic information; 3D view; checkCIF report

## Figures and Tables

**Table 1:** Hydrogen-bond geometry (Å, °)

*D*—H⋯*A*	*D*—H	H⋯*A*	*D*⋯*A*	*D*—H⋯*A*
N3—H3*A*⋯O5	0.86	1.80	2.659 (4)	174
C9—H9*A*⋯F1^i^	0.96	2.53	3.371 (5)	146
C10—H10*A*⋯O4^ii^	0.93	2.28	3.033 (5)	137
C1—H1*A*⋯O3^iii^	0.93	2.57	3.434 (5)	155
C11—H11*A*⋯O5^iii^	0.93	2.25	3.180 (5)	174

Symmetry codes: (i) 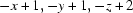; (ii) 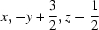; (iii) 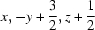.

**Fig. 1.**
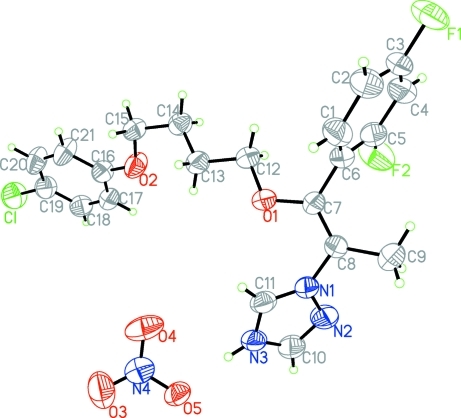
The molecular structure of the title compound, with the atom numbering scheme. Displacement ellipsoids are drawn at 30% probability levels.

**Fig. 2.**
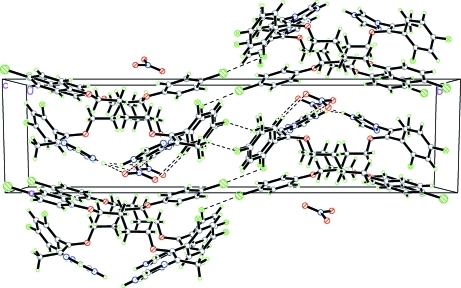
The packing diagram of the title compound. Hydron bonds are shown as dashed lines.

## supplementary crystallographic information

### Comment

Triazole derivatives such as voriconazole ((*2R,3S*)-2-(2,4-difluorophenyl) -3-(5-fluoropyrimidin-4-yl)-1-(1*H*-1,2,4-triazol-1-yl) butan-2-ol) and posaconazole (4-(4-(4-(4-(((*3R,5R*)-5-(2,4-difluorophenyl)-5-(1,2,4- triazol-1-ylmethyl)oxolan-3-yl)methoxy)phenyl)piperazin-1-yl)phenyl)- 2-((*2S,3S*)-2-hydroxypentan-3-yl)-1,2,4-triazol-3-one) are safe and effective antifungal agents. (Jeu *et al.*, 2003) As part of our studies on the synthesis of new triazole derivatives, the crystal structure of the title compound, having similar structure with omoconazol (Kurt *et al.*, 1987), was determined.

In the title compound (Fig. 1), the cation adopts a Z conformation about the C═C double bond. In the crystal structure the anions and cations are connected via N—H···O, C—H···O and C—H···F hydrogen bonding (Table 1 and Fig. 2).

### Experimental

1-(2,4-Difluorophenyl)-2-(1,2,4-triazol)-1-y1)propan-1-one (3.0 g, 0.01 mol) 10 g of a 50% aqueous sodium hydroxide, toluene (15 ml) and 1.5 ml of a 40% aqueous solution of tetrabutyl ammonium hydroxide were mixed and heated to 323 K under vigorous stirring. 1-Bromo-4-(4-chlorophenoxy)-butane (2.6 g, 0.01 mol) dissolved in 10 ml toluene, was instilled into the stirred and warmed solution in the course of 10 h. The mixture was subsequently stirred for another 20 h at 323 K. The reaction mixture was mixed with as much water and chloroform so that the aqueous phase becomes lighter than the organic phase. Thereafter, the organic and aqueous phases were separated. The organic phase was dried with sodium sulfate. The solvents were distilled under reduced pressure. The remaining residue was a dark oil that was diluted with 10 ml 2-propanol and then adjusted to a pH-value of 2 by 30% aqueous nitric acid. The resulting nitric acid solution was then cooled in the refrigerator. The impure precipitated product herein was subsequently crystallized from a 1:1 mixture of ethyl acetate and ethanol. The purified product was analytically identified as an approximately pure *Z*-isomer of the title compound. Crystals of the title compound suitable for X-ray diffraction were obtained by slow evaporation of an ethanol solution. Details on the synthesis can be found in the literature (Ludwig & Kurt, 1985).

### Refinement

H atoms were positioned geometrically with C—H = 0.93 and 0.97 Å for aromatic and methylene H atoms, respectively, and with N—H = 0.86 Å for triazole H atom, and constrained to ride on their parent atoms, with *U*_iso_(H) = 1.2 (or 1.5 for methyl groups) times *U*_eq_(C).

### Crystal data

**Table:**

C_21_H_21_ClF_2_N_3_O_2_^+^·NO_3_^−^	*F*(000) = 1000
*M**_r_* = 482.87	*D*_x_ = 1.398 Mg m^−^^3^
Monoclinic, *P*2_1_/*c*	Mo *K*α radiation, λ = 0.71073 Å
Hall symbol: -P 2ybc	Cell parameters from 25 reflections
*a* = 7.9580 (16) Å	θ = 9–12°
*b* = 31.635 (6) Å	µ = 0.22 mm^−^^1^
*c* = 9.1850 (18) Å	*T* = 293 K
β = 97.29 (3)°	Block, yellow
*V* = 2293.6 (8) Å^3^	0.30 × 0.10 × 0.10 mm
*Z* = 4	

### Data collection

**Table:**

Enraf–Nonius CAD-4 diffractometer	1952 reflections with *I* > 2σ(*I*)
Radiation source: fine-focus sealed tube	*R*_int_ = 0.092
graphite	θ_max_ = 25.4°, θ_min_ = 1.3°
ω/2θ scans	*h* = 0→9
Absorption correction: ψ scan (North *et al.*, 1968)	*k* = 0→38
*T*_min_ = 0.936, *T*_max_ = 0.978	*l* = −11→10
4521 measured reflections	3 standard reflections every 200 reflections
4216 independent reflections	intensity decay: 1%

### Refinement

**Table:**

Refinement on *F*^2^	Primary atom site location: structure-invariant direct methods
Least-squares matrix: full	Secondary atom site location: difference Fourier map
*R*[*F*^2^ > 2σ(*F*^2^)] = 0.066	Hydrogen site location: inferred from neighbouring sites
*wR*(*F*^2^) = 0.140	H-atom parameters constrained
*S* = 1.00	*w* = 1/[σ^2^(*F*_o_^2^) + (0.048*P*)^2^] where *P* = (*F*_o_^2^ + 2*F*_c_^2^)/3
4216 reflections	(Δ/σ)_max_ < 0.001
298 parameters	Δρ_max_ = 0.20 e Å^−^^3^
0 restraints	Δρ_min_ = −0.24 e Å^−^^3^

### Special details

**Table:**

Geometry. All e.s.d.'s (except the e.s.d. in the dihedral angle between two l.s. planes) are estimated using the full covariance matrix. The cell e.s.d.'s are taken into account individually in the estimation of e.s.d.'s in distances, angles and torsion angles; correlations between e.s.d.'s in cell parameters are only used when they are defined by crystal symmetry. An approximate (isotropic) treatment of cell e.s.d.'s is used for estimating e.s.d.'s involving l.s. planes.
Refinement. Refinement of *F*^2^ against ALL reflections. The weighted *R*-factor *wR* and goodness of fit *S* are based on *F*^2^, conventional *R*-factors *R* are based on *F*, with *F* set to zero for negative *F*^2^. The threshold expression of *F*^2^ > σ(*F*^2^) is used only for calculating *R*-factors(gt) *etc*. and is not relevant to the choice of reflections for refinement. *R*-factors based on *F*^2^ are statistically about twice as large as those based on *F*, and *R*- factors based on ALL data will be even larger.

### Fractional atomic coordinates and isotropic or equivalent isotropic displacement parameters (Å^2^)

**Table:**

	*x*	*y*	*z*	*U*_iso_*/*U*_eq_	
Cl	1.06795 (17)	0.98117 (4)	0.78129 (14)	0.0884 (4)	
O1	0.5208 (3)	0.66925 (8)	0.7812 (2)	0.0537 (7)	
N1	0.4023 (4)	0.64572 (10)	0.5034 (3)	0.0492 (8)	
F1	0.6286 (3)	0.51310 (8)	1.2146 (3)	0.0929 (9)	
C1	0.4331 (5)	0.59449 (12)	0.9814 (4)	0.0600 (11)	
H1A	0.3389	0.6121	0.9757	0.072*	
O2	0.8631 (4)	0.80660 (8)	0.8732 (3)	0.0665 (8)	
F2	0.7867 (3)	0.57220 (8)	0.7858 (3)	0.0912 (9)	
N2	0.4433 (5)	0.63841 (11)	0.3638 (3)	0.0667 (10)	
C2	0.4625 (6)	0.56669 (14)	1.0964 (4)	0.0680 (13)	
H2B	0.3904	0.5656	1.1684	0.082*	
N3	0.3111 (4)	0.69903 (10)	0.3756 (3)	0.0534 (9)	
H3A	0.2657	0.7229	0.3485	0.064*	
C3	0.5993 (7)	0.54084 (13)	1.1027 (4)	0.0622 (12)	
C4	0.7106 (6)	0.54183 (13)	0.9996 (4)	0.0668 (12)	
H4A	0.8045	0.5241	1.0054	0.080*	
C5	0.6748 (6)	0.57053 (13)	0.8877 (4)	0.0569 (11)	
C6	0.5399 (5)	0.59716 (12)	0.8733 (4)	0.0463 (10)	
C7	0.5057 (5)	0.62726 (12)	0.7499 (4)	0.0444 (9)	
C8	0.4462 (5)	0.61470 (11)	0.6153 (4)	0.0498 (10)	
C9	0.4189 (6)	0.57050 (13)	0.5668 (4)	0.0864 (16)	
H9A	0.4507	0.5518	0.6480	0.130*	
H9B	0.4867	0.5645	0.4899	0.130*	
H9C	0.3014	0.5664	0.5307	0.130*	
C10	0.3847 (6)	0.67162 (14)	0.2922 (4)	0.0652 (12)	
H10A	0.3928	0.6760	0.1932	0.078*	
C11	0.3222 (5)	0.68190 (12)	0.5088 (4)	0.0506 (10)	
H11A	0.2811	0.6934	0.5905	0.061*	
C12	0.6628 (5)	0.68174 (11)	0.8866 (4)	0.0484 (10)	
H12A	0.7679	0.6721	0.8546	0.058*	
H12B	0.6527	0.6691	0.9813	0.058*	
C13	0.6635 (5)	0.72830 (11)	0.8991 (4)	0.0528 (10)	
H13A	0.5584	0.7374	0.9325	0.063*	
H13B	0.6681	0.7405	0.8028	0.063*	
C14	0.8110 (5)	0.74505 (12)	1.0040 (4)	0.0503 (10)	
H14A	0.8013	0.7342	1.1014	0.060*	
H14B	0.9152	0.7339	0.9746	0.060*	
C15	0.8246 (5)	0.79196 (12)	1.0125 (4)	0.0542 (10)	
H15A	0.7186	0.8041	1.0342	0.065*	
H15B	0.9135	0.8002	1.0895	0.065*	
C16	0.9051 (5)	0.84798 (12)	0.8603 (4)	0.0526 (10)	
C17	0.9642 (5)	0.85865 (13)	0.7305 (4)	0.0591 (11)	
H17A	0.9710	0.8380	0.6593	0.071*	
C18	1.0133 (5)	0.89934 (14)	0.7049 (4)	0.0617 (11)	
H18A	1.0538	0.9062	0.6172	0.074*	
C19	1.0020 (5)	0.93006 (13)	0.8108 (5)	0.0617 (11)	
C20	0.9427 (6)	0.91986 (14)	0.9391 (5)	0.0724 (13)	
H20A	0.9355	0.9405	1.0100	0.087*	
C21	0.8929 (5)	0.87874 (13)	0.9644 (4)	0.0667 (12)	
H21A	0.8514	0.8720	1.0517	0.080*	
N4	0.1836 (4)	0.79947 (13)	0.3777 (4)	0.0604 (9)	
O3	0.1244 (4)	0.83434 (11)	0.3510 (4)	0.0941 (11)	
O4	0.2476 (4)	0.78801 (11)	0.5018 (3)	0.0883 (11)	
O5	0.1781 (3)	0.77208 (8)	0.2747 (3)	0.0588 (7)	

### Atomic displacement parameters (Å^2^)

**Table:**

	*U*^11^	*U*^22^	*U*^33^	*U*^12^	*U*^13^	*U*^23^
Cl	0.1080 (10)	0.0611 (8)	0.0955 (9)	−0.0204 (7)	0.0108 (7)	0.0053 (7)
O1	0.0688 (19)	0.0470 (16)	0.0404 (14)	−0.0018 (13)	−0.0116 (13)	−0.0013 (12)
N1	0.066 (2)	0.046 (2)	0.0340 (17)	0.0003 (17)	−0.0015 (15)	0.0002 (15)
F1	0.126 (2)	0.0812 (19)	0.0663 (16)	−0.0072 (17)	−0.0075 (15)	0.0369 (14)
C1	0.075 (3)	0.054 (3)	0.052 (2)	0.003 (2)	0.011 (2)	0.010 (2)
O2	0.102 (2)	0.0489 (18)	0.0517 (17)	−0.0183 (16)	0.0205 (15)	−0.0070 (14)
F2	0.090 (2)	0.106 (2)	0.0833 (18)	0.0335 (17)	0.0354 (16)	0.0291 (15)
N2	0.095 (3)	0.072 (3)	0.0321 (18)	0.014 (2)	0.0053 (18)	−0.0024 (17)
C2	0.094 (4)	0.066 (3)	0.047 (3)	−0.008 (3)	0.021 (2)	0.011 (2)
N3	0.067 (2)	0.051 (2)	0.0388 (18)	−0.0046 (18)	−0.0070 (16)	0.0064 (16)
C3	0.098 (4)	0.045 (3)	0.040 (2)	−0.009 (3)	−0.006 (2)	0.0116 (19)
C4	0.082 (3)	0.061 (3)	0.054 (3)	0.016 (2)	−0.004 (2)	0.007 (2)
C5	0.077 (3)	0.056 (3)	0.039 (2)	0.001 (2)	0.011 (2)	−0.001 (2)
C6	0.058 (3)	0.045 (2)	0.034 (2)	0.002 (2)	0.0029 (19)	0.0002 (17)
C7	0.051 (2)	0.039 (2)	0.041 (2)	−0.0049 (18)	−0.0012 (17)	−0.0023 (17)
C8	0.069 (3)	0.040 (2)	0.038 (2)	−0.001 (2)	−0.0001 (19)	0.0038 (18)
C9	0.132 (5)	0.057 (3)	0.062 (3)	−0.017 (3)	−0.022 (3)	−0.003 (2)
C10	0.093 (4)	0.066 (3)	0.035 (2)	0.001 (3)	0.000 (2)	0.001 (2)
C11	0.059 (3)	0.054 (3)	0.036 (2)	−0.007 (2)	−0.0015 (18)	0.0002 (19)
C12	0.049 (2)	0.051 (2)	0.043 (2)	−0.002 (2)	−0.0027 (18)	−0.0036 (18)
C13	0.063 (3)	0.047 (3)	0.047 (2)	−0.006 (2)	−0.0001 (19)	−0.0017 (19)
C14	0.045 (2)	0.057 (3)	0.048 (2)	−0.001 (2)	0.0024 (19)	−0.0049 (19)
C15	0.057 (3)	0.056 (3)	0.048 (2)	−0.003 (2)	−0.0002 (19)	−0.008 (2)
C16	0.057 (3)	0.050 (3)	0.051 (2)	−0.008 (2)	0.010 (2)	0.000 (2)
C17	0.067 (3)	0.054 (3)	0.056 (3)	−0.002 (2)	0.005 (2)	−0.008 (2)
C18	0.063 (3)	0.066 (3)	0.057 (3)	−0.004 (2)	0.010 (2)	0.003 (2)
C19	0.069 (3)	0.050 (3)	0.066 (3)	−0.011 (2)	0.006 (2)	0.001 (2)
C20	0.093 (4)	0.052 (3)	0.075 (3)	−0.014 (3)	0.022 (3)	−0.020 (2)
C21	0.087 (3)	0.059 (3)	0.059 (3)	−0.024 (2)	0.030 (2)	−0.018 (2)
N4	0.055 (2)	0.075 (3)	0.053 (2)	−0.007 (2)	0.0119 (18)	−0.002 (2)
O3	0.089 (3)	0.062 (2)	0.132 (3)	0.011 (2)	0.014 (2)	−0.007 (2)
O4	0.097 (2)	0.130 (3)	0.0366 (17)	−0.010 (2)	0.0009 (16)	−0.0060 (18)
O5	0.077 (2)	0.0595 (18)	0.0377 (14)	−0.0015 (15)	0.0004 (13)	−0.0056 (14)

### Geometric parameters (Å, °)

**Table:**

Cl—C19	1.732 (4)	C9—H9C	0.9600
O1—C7	1.361 (4)	C10—H10A	0.9300
O1—C12	1.447 (4)	C11—H11A	0.9300
N1—C11	1.314 (4)	C12—C13	1.477 (4)
N1—N2	1.383 (4)	C12—H12A	0.9700
N1—C8	1.432 (4)	C12—H12B	0.9700
F1—C3	1.349 (4)	C13—C14	1.517 (5)
C1—C2	1.371 (5)	C13—H13A	0.9700
C1—C6	1.389 (5)	C13—H13B	0.9700
C1—H1A	0.9300	C14—C15	1.489 (5)
O2—C16	1.360 (4)	C14—H14A	0.9700
O2—C15	1.430 (4)	C14—H14B	0.9700
F2—C5	1.372 (4)	C15—H15A	0.9700
N2—C10	1.295 (5)	C15—H15B	0.9700
C2—C3	1.357 (6)	C16—C21	1.376 (5)
C2—H2B	0.9300	C16—C17	1.378 (5)
N3—C11	1.330 (4)	C17—C18	1.374 (5)
N3—C10	1.340 (5)	C17—H17A	0.9300
N3—H3A	0.8600	C18—C19	1.386 (5)
C3—C4	1.377 (6)	C18—H18A	0.9300
C4—C5	1.374 (5)	C19—C20	1.362 (5)
C4—H4A	0.9300	C20—C21	1.388 (5)
C5—C6	1.358 (5)	C20—H20A	0.9300
C6—C7	1.479 (5)	C21—H21A	0.9300
C7—C8	1.327 (5)	N4—O3	1.212 (4)
C8—C9	1.475 (5)	N4—O4	1.243 (4)
C9—H9A	0.9600	N4—O5	1.279 (4)
C9—H9B	0.9600		
			
C7—O1—C12	116.6 (3)	O1—C12—C13	108.6 (3)
C11—N1—N2	110.7 (3)	O1—C12—H12A	110.0
C11—N1—C8	130.2 (3)	C13—C12—H12A	110.0
N2—N1—C8	119.1 (3)	O1—C12—H12B	110.0
C2—C1—C6	122.0 (4)	C13—C12—H12B	110.0
C2—C1—H1A	119.0	H12A—C12—H12B	108.3
C6—C1—H1A	119.0	C12—C13—C14	113.1 (3)
C16—O2—C15	118.2 (3)	C12—C13—H13A	109.0
C10—N2—N1	102.9 (3)	C14—C13—H13A	109.0
C3—C2—C1	118.5 (4)	C12—C13—H13B	109.0
C3—C2—H2B	120.8	C14—C13—H13B	109.0
C1—C2—H2B	120.8	H13A—C13—H13B	107.8
C11—N3—C10	106.2 (3)	C15—C14—C13	115.2 (3)
C11—N3—H3A	126.9	C15—C14—H14A	108.5
C10—N3—H3A	126.9	C13—C14—H14A	108.5
F1—C3—C2	119.0 (4)	C15—C14—H14B	108.5
F1—C3—C4	118.3 (4)	C13—C14—H14B	108.5
C2—C3—C4	122.7 (4)	H14A—C14—H14B	107.5
C5—C4—C3	116.0 (4)	O2—C15—C14	107.3 (3)
C5—C4—H4A	122.0	O2—C15—H15A	110.2
C3—C4—H4A	122.0	C14—C15—H15A	110.2
C6—C5—F2	118.9 (3)	O2—C15—H15B	110.2
C6—C5—C4	124.7 (4)	C14—C15—H15B	110.2
F2—C5—C4	116.4 (4)	H15A—C15—H15B	108.5
C5—C6—C1	116.1 (4)	O2—C16—C21	125.5 (4)
C5—C6—C7	122.7 (3)	O2—C16—C17	115.2 (3)
C1—C6—C7	121.2 (4)	C21—C16—C17	119.3 (4)
C8—C7—O1	120.0 (3)	C18—C17—C16	120.9 (4)
C8—C7—C6	122.0 (3)	C18—C17—H17A	119.5
O1—C7—C6	117.6 (3)	C16—C17—H17A	119.5
C7—C8—N1	119.3 (3)	C17—C18—C19	119.4 (4)
C7—C8—C9	125.9 (3)	C17—C18—H18A	120.3
N1—C8—C9	114.8 (3)	C19—C18—H18A	120.3
C8—C9—H9A	109.5	C20—C19—C18	120.1 (4)
C8—C9—H9B	109.5	C20—C19—Cl	120.1 (3)
H9A—C9—H9B	109.5	C18—C19—Cl	119.8 (3)
C8—C9—H9C	109.5	C19—C20—C21	120.3 (4)
H9A—C9—H9C	109.5	C19—C20—H20A	119.9
H9B—C9—H9C	109.5	C21—C20—H20A	119.9
N2—C10—N3	113.0 (4)	C16—C21—C20	120.0 (4)
N2—C10—H10A	123.5	C16—C21—H21A	120.0
N3—C10—H10A	123.5	C20—C21—H21A	120.0
N1—C11—N3	107.2 (3)	O3—N4—O4	123.7 (4)
N1—C11—H11A	126.4	O3—N4—O5	119.4 (4)
N3—C11—H11A	126.4	O4—N4—O5	116.9 (4)
			
C11—N1—N2—C10	0.3 (4)	N2—N1—C8—C7	139.3 (4)
C8—N1—N2—C10	179.2 (3)	C11—N1—C8—C9	137.7 (4)
C6—C1—C2—C3	−0.8 (6)	N2—N1—C8—C9	−40.9 (5)
C1—C2—C3—F1	−179.4 (4)	N1—N2—C10—N3	0.2 (5)
C1—C2—C3—C4	0.9 (7)	C11—N3—C10—N2	−0.6 (5)
F1—C3—C4—C5	179.8 (3)	N2—N1—C11—N3	−0.7 (4)
C2—C3—C4—C5	−0.5 (6)	C8—N1—C11—N3	−179.4 (3)
C3—C4—C5—C6	0.0 (6)	C10—N3—C11—N1	0.8 (4)
C3—C4—C5—F2	179.0 (3)	C7—O1—C12—C13	176.6 (3)
F2—C5—C6—C1	−178.8 (3)	O1—C12—C13—C14	−178.3 (3)
C4—C5—C6—C1	0.2 (6)	C12—C13—C14—C15	175.8 (3)
F2—C5—C6—C7	1.6 (6)	C16—O2—C15—C14	−171.0 (3)
C4—C5—C6—C7	−179.4 (4)	C13—C14—C15—O2	−66.4 (4)
C2—C1—C6—C5	0.3 (6)	C15—O2—C16—C21	−8.9 (6)
C2—C1—C6—C7	179.8 (4)	C15—O2—C16—C17	171.0 (3)
C12—O1—C7—C8	−145.4 (4)	O2—C16—C17—C18	−179.0 (4)
C12—O1—C7—C6	41.0 (4)	C21—C16—C17—C18	1.0 (6)
C5—C6—C7—C8	74.0 (5)	C16—C17—C18—C19	−0.5 (6)
C1—C6—C7—C8	−105.6 (5)	C17—C18—C19—C20	0.1 (6)
C5—C6—C7—O1	−112.6 (4)	C17—C18—C19—Cl	178.6 (3)
C1—C6—C7—O1	67.8 (5)	C18—C19—C20—C21	−0.2 (7)
O1—C7—C8—N1	1.5 (6)	Cl—C19—C20—C21	−178.7 (4)
C6—C7—C8—N1	174.8 (3)	O2—C16—C21—C20	178.9 (4)
O1—C7—C8—C9	−178.3 (4)	C17—C16—C21—C20	−1.1 (6)
C6—C7—C8—C9	−5.0 (7)	C19—C20—C21—C16	0.7 (7)
C11—N1—C8—C7	−42.1 (6)		

### Hydrogen-bond geometry (Å, °)

**Table:**

*D*—H···*A*	*D*—H	H···*A*	*D*···*A*	*D*—H···*A*
N3—H3A···O5	0.86	1.80	2.659 (4)	174
C9—H9A···F1^i^	0.96	2.53	3.371 (5)	146
C10—H10A···O4^ii^	0.93	2.28	3.033 (5)	137
C1—H1A···O3^iii^	0.93	2.57	3.434 (5)	155
C11—H11A···O5^iii^	0.93	2.25	3.180 (5)	174

Symmetry codes: (i) −*x*+1, −*y*+1, −*z*+2; (ii) *x*, −*y*+3/2, *z*−1/2; (iii) *x*, −*y*+3/2, *z*+1/2.

## References

[bb1] Enraf–Nonius (1994). *CAD-4 EXPRES*S. Enraf–Nonius, Delft, The Netherlands.

[bb2] Harms, K. & Wocadlo, S. (1995). *XCAD4* University of Marburg, Germany.

[bb3] Jeu, L., Piacenti, F. J., Lyakhovetskiy, A. G. & Fung, H. B. (2003). *Clin. Ther.* **25**, 1321–1381. 10.1016/s0149-2918(03)80126-1 12867215

[bb4] Kurt, T., Ludwing, Z., Max, H. P., Martin, E. & Max, D. (1987). *Helv. Chim. Acta* **70**, 441–444.

[bb5] Ludwig, Z. & Kurt, T. (1985). US Patent Appl. US4554356.

[bb6] North, A. C. T., Phillips, D. C. & Mathews, F. S. (1968). *Acta Cryst.* A**24**, 351–359.

[bb7] Sheldrick, G. M. (2008). *Acta Cryst.* A**64**, 112–122. 10.1107/S0108767307043930 18156677

[bb8] Spek, A. L. (2009). *Acta Cryst.* D**65**, 148–155. 10.1107/S090744490804362X PMC2631630 19171970

